# Rab14 and Its Exchange Factor FAM116 Link Endocytic Recycling and Adherens Junction Stability in Migrating Cells

**DOI:** 10.1016/j.devcel.2012.04.010

**Published:** 2012-05-15

**Authors:** Andrea Linford, Shin-ichiro Yoshimura, Ricardo Nunes Bastos, Lars Langemeyer, Andreas Gerondopoulos, Daniel J. Rigden, Francis A. Barr

**Affiliations:** 1 Department of Biochemistry, University of Oxford, South Parks Road, Oxford OX1 3QU, UK; 2 University of Liverpool, Institute of Integrative Biology, Crown Street, Liverpool L69 7ZB, UK

## Abstract

Rab GTPases define the vesicle trafficking pathways underpinning cell polarization and migration. Here, we find that Rab4, Rab11, and Rab14 and the candidate Rab GDP-GTP exchange factors (GEFs) FAM116A and AVL9 are required for cell migration. Rab14 and its GEF FAM116A localize to and act on an intermediate compartment of the transferrin-recycling pathway prior to Rab11 and after Rab5 and Rab4. This Rab14 intermediate recycling compartment has specific functions in migrating cells discrete from early and recycling endosomes. Rab14-depleted cells show increased N-cadherin levels at junctional complexes and cannot resolve cell-cell junctions. This is due to decreased shedding of cell-surface N-cadherin by the ADAM family protease ADAM10/Kuzbanian. In FAM116A- and Rab14-depleted cells, ADAM10 accumulates in a transferrin-positive endocytic compartment, and the cell-surface level of ADAM10 is correspondingly reduced. FAM116 and Rab14 therefore define an endocytic recycling pathway needed for ADAM protease trafficking and regulation of cell-cell junctions.

## Graphical abstract

**Graphical Abstract:**

**Figure:**
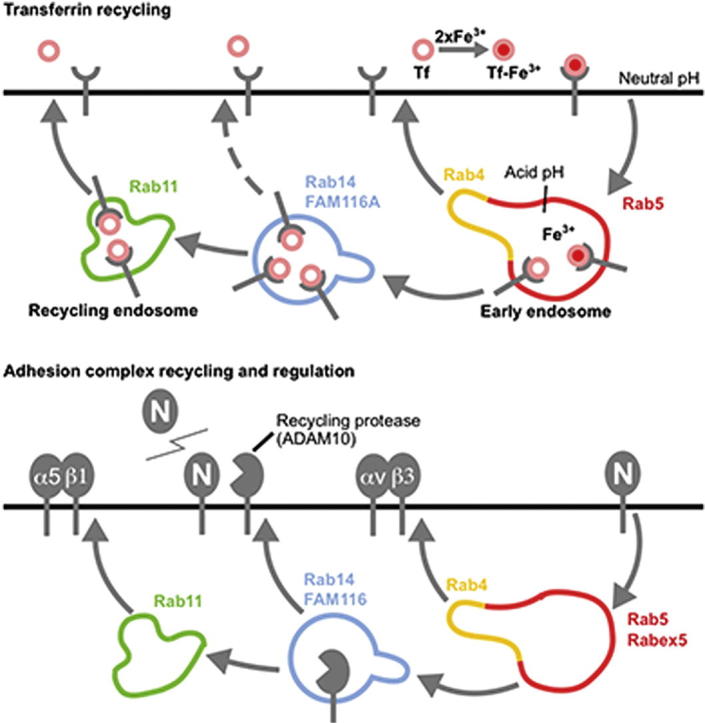

**Highlights:**

► FAM116A is a GDP-GTP exchange factor for the GTPase Rab14 ► Rab14 defines an endocytic recycling pathway required for ADAM10 transport ► In Rab14-depleted cells, ADAM10 fails to degrade its substrate, N-cadherin ► Dysregulation of ADAM10/N-cadherin accounts for Rab14 effects on cell migration

## Teaser

Linford et al. show that Rab14 and its exchange factor FAM116 are required for the endocytic recycling of the ADAM10 protease. In cells lacking Rab14 or FAM116, ADAM10 is mislocalized and cannot process its substrate N-cadherin. This leads to stabilization of the adherens junctions and thereby interferes with cell migration.

## Introduction

Cell migration and polarization are underpinned by a complex network of cellular trafficking pathways, which transport a variety of different membrane proteins required for sensing extracellular cues, as well as the creation and remodeling of cell adhesions and cell-cell junctions (Baum and Georgiou, 2011; Caswell et al., 2009; Huttenlocher, 2005; Mellman and Nelson, 2008; Ulrich and Heisenberg, 2009). In addition to their physiological functions during development, altered cell polarization and increased cell migration are also found in disease states, such as cancer, or in response to physical wounds, which require coordinated changes in cell polarization, proliferation, and cell migration if the damage is to be repaired (Caswell et al., 2009; Mellman and Nelson, 2008).

In recent years, Rab-GTPase-directed endocytic trafficking pathways have begun to emerge as key transport events required for remodelling of cell adhesion and cellular junctions, as well as cell polarization and migration (Baum and Georgiou, 2011; Caswell et al., 2009). Rab5-dependent endocytic transport of E- and N-cadherins is important for controlling cell-cell adhesions during vertebrate gastrulation and brain development (Kawauchi et al., 2010; Ulrich et al., 2005), as well as receptor trafficking during the accompanying signaling events (Assaker et al., 2010; Jékely et al., 2005). The Rab5-related GTPase Rab21 also functions in β1-integrin trafficking and cell invasion (Hooper et al., 2010; Mai et al., 2011; Pellinen et al., 2006). Transport of signaling receptors required for proper axonal guidance requires Rab27 (Arimura et al., 2009). Best understood are probably the Rab11 family of GTPases, Rab4, Rab11, and Rab25 that play key roles in the transport of different integrin complexes in migrating cells (Caswell et al., 2007, 2009), E-cadherin trafficking during adherens junction formation (Le et al., 1999; Lock and Stow, 2005), and in cell polarization during asymmetric cell divisions (Emery et al., 2005). Rab11 family GTPases are typically associated with different stages of the endocytic recycling pathway (de Renzis et al., 2002; Sönnichsen et al., 2000; Ullrich et al., 1996), for which the transferrin receptor is the most studied cargo.

Although endocytic recycling is often viewed as a single pathway, this may not be the case, because specific Rabs are associated with the trafficking of different cargo molecules to discrete regions of the cell surface. Accordingly, Rab11 and its effector, the Rab-coupling protein (RCP), promote α5β1-integrin recycling (Caswell et al., 2008), whereas Rab25 makes direct contact to the cytoplasmic tail of α5β1-integrin and promotes its delivery to the tips of elongated pseudohyphae during invasive cell migration in three-dimensional (3D) matrices (Caswell et al., 2007). Rab4 promotes the recycling of αVβ3-integrin and cell migration through the RUN (RPIP8/UNIC-14, NESCA) and FYVE (FAB1/YOTB/VAC1/EEA1) domain effector protein RUFY1 (Roberts et al., 2001; Vukmirica et al., 2006). Rab14, the final member of the Rab11 subfamily has not been directly linked to cell migration events or the traffic of a specific cargo, although it is known to interact with both RCP and RUFY1 (Kelly et al., 2010; Yamamoto et al., 2010) and was identified in the proteome of endosomes isolated from migrating cells together with Rab5, Rab7, and Rab11 (Howes et al., 2010).

A crucial component of Rab function is the requirement for activation at a specific membrane surface (Pfeffer and Aivazian, 2004; Zerial and McBride, 2001). This is achieved by specific guanine nucleotide exchange factors (GEFs) that promote the release of GDP and binding of GTP (Barr and Lambright, 2010). The known Rab GEFs typically fall into discrete families defined by a number of conserved, yet structurally unrelated protein domains and protein complexes (Barr and Lambright, 2010; Wu et al., 2011). However, to date, none of these GEF families has been definitively shown to act on the Rab11 GTPases, Rab4, Rab11, Rab14, and Rab25 or to play an essential role in processes controlled by these GTPases. We therefore set out to identify candidate Rab-Rab GEF pathways required for cell migration in vitro and assign the underlying membrane trafficking pathways and order they act in.

## Results

### Rabs Required for Cell Migration In Vitro

**Figure 1:**
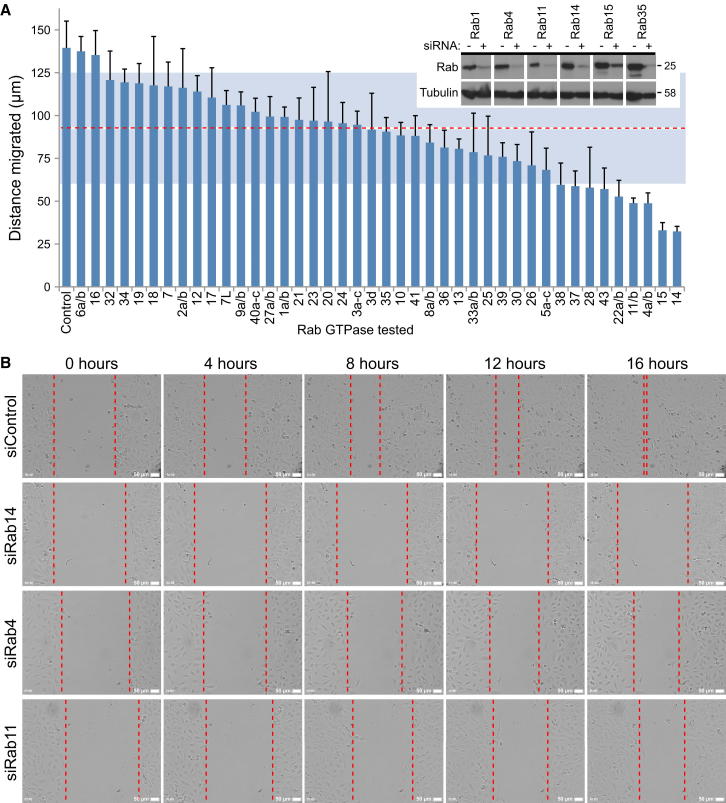
Rab14 Is Required for Cell Migration In Vitro (A) A549 cells in 24-well plates were treated with siRNA duplexes targeting the human Rab GTPases family. Similar Rabs were pooled, see bar graph legend, because of concerns that they may be functionally redundant. After 72 hr, the cell monolayers were wounded and then after a 30 min recovery period were imaged for 16 hr. The distance migrated by the wound front after 16 hr is plotted in the bar graph. Error bars indicate the standard deviation (n = 3). The red dotted line indicates the median distance migrated, and the light blue-gray shading outlines the boundaries of the standard deviation for all conditions. To test for depletion, efficiency cells were treated with siRNA control duplex (-) or siRNA duplexes for the Rab GTPases. After 72 hr, the cells were lysed in SDS-PAGE sample buffer and then western blotted as indicated in the panel inset in the graph. Tubulin was used as a loading control. (B) Brightfield images from control, FAM116A-depleted, and Avl9-depleted cells are shown at 0, 4, 8, 12, and 16 hr. The dotted red lines mark the edges of the wound at each time point. The scale bars indicate 50 μm.

Confluent monolayers of A549 lung epithelial cells, grown to create a single sheet of cells linked by adherens junctions, were scratched using a plastic pipette tip. This creates a wound or break in the cell monolayer into which the adjacent cells can migrate. The migration event is associated with cell polarization in the direction of the wound and loss of junctions with neighboring cells. Once the wound has closed, the cells reform a confluent sheet-like monolayer. To identify trafficking pathways required for cell migration in vitro, this scratch-wound cell migration assay was combined with siRNA depletion of human Rab GTPases (Figure 1). Because of concerns that some closely related Rabs may be redundant, these were combined into one pool for the purposes of the screen. Measurements of cell migration after 16 hr revealed that depletion of most Rabs resulted in reduced cell migration relative to the negative control (Figure 1A). It was therefore decided to focus on the most severe migration defects, defined as at least one standard deviation under the median migration distance (Figure 1A, dotted red line marks the median, shaded light blue-gray area the standard deviation; Movie S1 available online). Using these criteria, Rab4, Rab11, Rab14, and Rab15 were found to have a migration defect. Rab14-depleted cells remained as a sheet and only spread slightly into the wounded area (Figure 1B and Movie S2). Consistent with previous studies showing that Rab4 and Rab11 are required for integrin and E-cadherin trafficking, Rab4- and Rab11-depleted cells migrated more slowly away from the edge of the cell sheet and failed to close the wound after 16 hr (Figure 1B). Interestingly, Rab4, Rab11, and Rab14 fall into the same Rab11 subfamily and have all been implicated in endocytic recycling (Ullrich et al., 1996; Yamamoto et al., 2010). However, because all three were identified in this screen, it is unlikely that they have redundant functions, suggesting that they regulate the transport of specific cargo molecules. Simultaneous depletion of the three Rab5 isoforms resulted in a 50% reduction in migration relative to the control; however, this was slightly above the threshold used (Figure 1A). This is possibly a false negative due to incomplete depletion of all three forms of Rab5, which is needed to block endocytosis of cargo, such as the EGF receptor (Fuchs et al., 2007). Rab15 was also identified in this screen, but this was not investigated further at this time.

### Rab Regulatory Pathways Required for Cell Migration In Vitro

**Figure 2:**
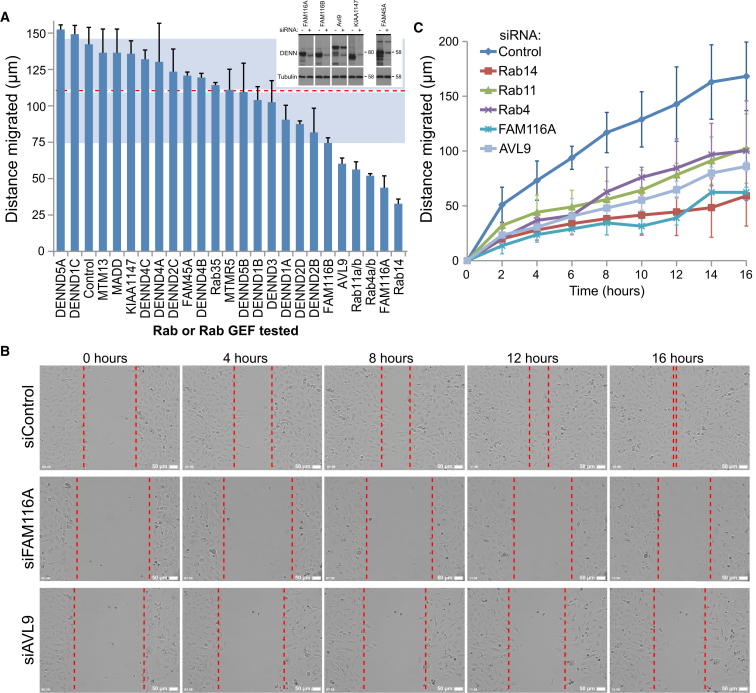
The DENN-Related Proteins FAM116 and Avl9 Are Required for Cell Migration In Vitro (A) A549 cells in 24-well plates were treated with siRNA duplexes targeting the human DENN and DENN-related proteins described in Figure S1. After 72 hr, the cell monolayers were wounded and then after a 30 min recovery period were imaged for 16 hr. The distance migrated by the wound front after 16 hr is plotted in the bar graph. Error bars indicate the standard deviation (n = 3). The red dotted line indicates the median distance migrated, and the light blue-gray shading outlines the boundaries of the standard deviation for all conditions. To test for depletion, efficiency cells were treated with siRNA control duplex (-) or siRNA duplexes for the DENN-related proteins. After 72 hr, the cells were lysed in SDS-PAGE sample buffer and then western blotted as indicated in the panel inset in the graph. Tubulin was used as a loading control. (B) Brightfield images from control, FAM116A-depleted, and Avl9-depleted cells are shown at 0, 4, 8, 12, and 16 hr. The scale bars indicate 50 μm. (C) A549 cells in 24-well plates were treated with siRNA duplexes targeting Rab4, Rab11, Rab14, FAM116A, and Avl9. After 72 hr, the cell monolayers were wounded and then after a 30 min recovery period were imaged for 16 hr. The distance migrated by the wound front is plotted over time in the graph; error bars show the standard deviation (n = 3).

To further define the trafficking pathways involved in cell migration, the effects of depleting known and candidate Rab GEFs were investigated. At present, the cellular GEFs for Rab4, Rab11, and Rab14 are unknown. Recent evidence shows that the differentially expressed in normal and neoplasia (DENN) domain proteins form a large family of Rab GEFs (Allaire et al., 2010; Levivier et al., 2001; Sato et al., 2008; Yoshimura et al., 2010); however, none have been reported to act on Rab11-related GTPases. In addition to the core DENN domain proteins, there is a small group of proteins with DENN-related domains FAM116A, FAM116B, Avl9, FAM45A, and KIAA1147 (Figure S1). Of these, FAM116 proteins show the highest degree of sequence similarity to the DENN domain and have been referred to as DENND6 (Marat et al., 2011). Although there is some evidence that budding yeast Avl9 is not a Rab GEF, there is no data on the specificity of the other DENN-related proteins. To test if any of the DENN or DENN-related proteins were required for A549 cell migration, they were depleted, and scratch-wound cell migration assays were performed (Figure 2). This approach showed that FAM116A-, FAM116B-, and Avl9-depleted cells showed migration defects comparable to the Rab11 subfamily (Figure 2A). FAM116A-depleted cells remained as a sheet and only spread slightly into the wounded area (Figure 2B and Movie S3). Similarly, Avl9-depleted cells migrated more slowly and failed to close the wound after 16 hr (Figure 2B and Movie S3). Comparison of distance migrated over time indicated that FAM116A and Rab14 showed the strongest migration defect (Figure 2C). Rab4, Rab11, and Avl9 all migrated more slowly than the control but moved further than FAM116A- or Rab14-depleted cells (Figure 2C). The DENN-related candidate Rab GEFs FAM116A and Avl9 therefore function during cell migration events, possibly by controlling the Rab11 subfamily of GTPases.

### FAM116 Proteins Have Rab14 GEF Activity

**Figure 3:**
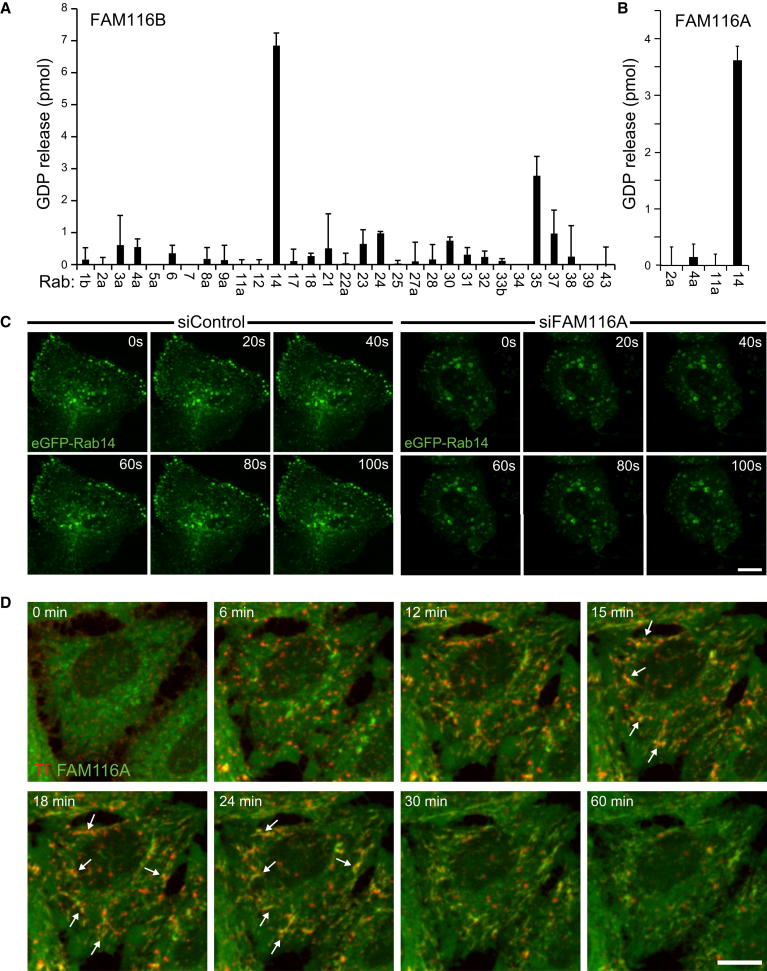
FAM116 Proteins Have GEF Activity toward Rab14 (A and B) Human FAM116A and FAM116B were tested against a representative panel of human Rab proteins using the GDP-releasing assay. Briefly, 10 μg of each GST-tagged Rab to be tested was incubated in 50 mM HEPES-NaOH (pH 6.8), 0.1 mg/ml BSA, 125 μM EDTA, 10 μM Mg^2+^-GDP, and 5 μCi [^3^H]-GDP (10 mCi/ml; 5000 Ci/mmol) in a total volume of 200 μl for 15 min at 30°C to load the Rab with the radioactive GDP probe. For standard GDP-releasing GEF assays, 100 μl of the loading reaction was then mixed with 10 μl 10 mM Mg^2+^-GTP, 10 nM Hexahistidine-tagged FAM116A purified from bacteria or a buffer control, and adjusted to 120 μl final volume with assay buffer. The GEF reaction occurred for 20 min at 30°C. After this, 2.5 μl were taken for a specific activity measurement; the remainder was split into two tubes and then incubated with 500 μl ice-cold assay buffer containing 1 mM MgCl_2_ and 20 μl packed glutathione-sepharose for 60 min at 4°C to separate Rab-GDP complexes from free “released” GDP. After washing three times with 500 μl ice-cold assay buffer, the sepharose was transferred to a vial containing 4 ml scintillation fluid and counted. The amount of nucleotide exchange was calculated in pmoles GDP-released. Error bars show the standard error (n = 4). (C) HeLa cells were treated with control or FAM116A siRNA duplexes for 48 hr and then transfected with eGFP-Rab14 expression constructs. After a further 24 hr, the cells were imaged every 20 s using a spinning disk confocal microscope. A maximum intensity projection of the eGFP signal in the entire cell volume is shown. (D) HeLa cells expressing eGFP-FAM116A were used for live cell imaging of transferrin uptake assays. Maximum intensity projection images of the entire cell volume are shown for the time points indicated. Arrows mark regions over colocalization at 15–24 min. The scale bar indicates 10 μm in all panels. See also Figure S2.

To identify the targets of FAM116A, FAM116B, and Avl9, GDP-GTP exchange factor assays were carried out using purified recombinant proteins. Despite repeated attempts, it was not possible to measure Avl9 GEF activity toward any of the Rabs tested under the assay conditions used. By contrast, FAM116B displayed activity toward Rab14 and, to a lesser extent, Rab35 (Figure 3A). No measureable activity toward Rab4 and Rab11 was observed. Similarly, FAM116A was only active toward Rab14 when tested against the Rab11 subfamily (Figure 3B). Together, these findings support the assignment of FAM116 proteins as physiological GEFs for Rab14.

### FAM116 Is Required for Rab14 Recruitment to Recycling Endosomes

Rab GEFs play a crucial role in Rab activation at membrane surfaces, and loss of Rab GEF function is predicted to result in altered Rab localization. To test this, cells expressing eGFP-Rab14 were imaged every 20 s. In control cells, Rab14 was observed at small slowly moving punctae collected at the cell periphery, with some additional signal spread throughout the cytoplasm (Figure 3C). This localization was strikingly altered in FAM116A-depleted cells, where eGFP-Rab14 accumulated on large ring-like structures adjacent to the nucleus (Figure 3C), which stained positive for the transferrin receptor (Figure S2A). The clustering of enlarged Rab14 and transferrin receptor positive structures close to the nucleus is consistent with the report that Rab14 promotes microtubule-dependent trafficking to the cell surface (Ueno et al., 2011). By contrast, there was no difference in the behavior of eGFP-Rab11 in control and FAM116A-depleted cells (data not shown). FAM116 proteins therefore have GEF activity toward Rab14 in vitro, and depletion of FAM116A results in altered localization of eGFP-Rab14 in living cells.

To confirm where FAM116A acts, cells expressing FAM116A were stained for markers to different cellular organelles. This revealed that FAM116A was present in the cytoplasm and fine membrane tubules that overlapped with a population of the transferrin receptor, a recycling endosome marker, but not markers for early or late endosomes, lysosomes, or the Golgi apparatus (Figure S2B). Avl9 also showed some overlap with the transferrin receptor positive recycling endosomes but not early endosome markers (Figure S2B). However, unlike FAM116A expression, Avl9 did not trigger tubulation of the transferrin receptor staining, consistent with the idea that Avl9 and FAM116 act on different target GTPases. Live cell imaging of transferrin uptake was then performed on eGFP-FAM116A expressing cells (Figure 3D). Transferrin bound to its receptor had a diffuse, finely punctate staining pattern at the cell surface and then rapidly coalesced into larger punctate structures, expected to be early endosomes, within 6 min (Figure 3D, red signal). FAM116A was present on a dynamic tubular-reticular compartment and also gave a diffuse cytoplasmic staining (Figure 3D, green signal). From 12 to 24 min of uptake when transferrin is passing through the recycling endosomes, the transferrin signal overlapped with the tubular component of FAM116A (Figure 3D, yellow signal marked with arrows; Movie S4). This overlap diminished after 30 min, and the transferrin signal was lost after 60 min, as expected if it recycled to the cell surface (Figure 3D). Together, these findings support the idea that FAM116A and Rab14 localize to an endocytic recycling compartment on the transferrin uptake pathway.

### Rab14 and FAM116 Function at an Intermediate Step of the Transferrin Recycling Pathway

**Figure 4:**
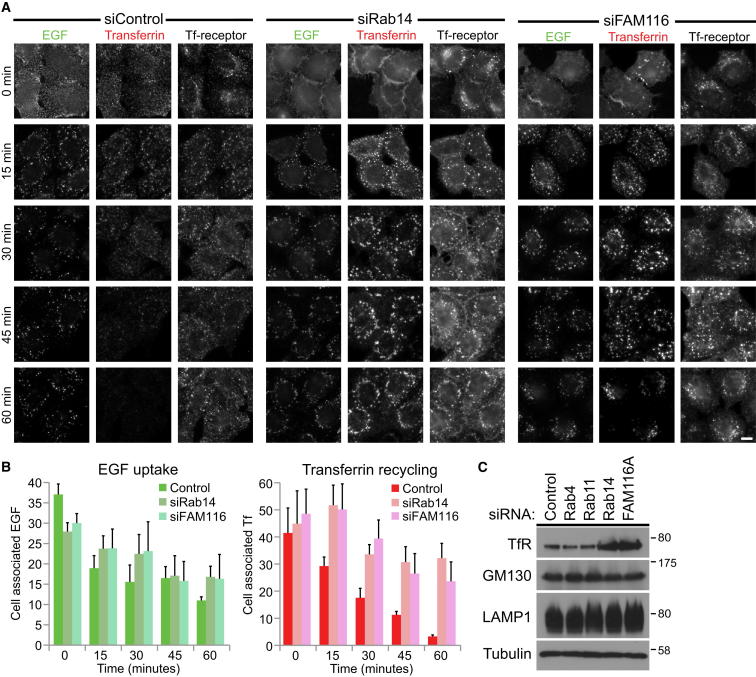
FAM116 and Rab14 Act on the Transferrin Receptor Recycling Pathway (A) HeLa cells were treated with control, Rab14, or FAM116A siRNA duplexes for 72 hr. The cells were then used for combined transferrin and EGF uptake assays. Samples were fixed at 0, 15, 30, 45, and 60 min and then stained with antibodies to the transferrin receptor and DAPI. The scale bar indicates 10 μm. (B) The amount of cell-associated EGF and transferrin was measured using ImageJ for both control and Rab14-depleted cells and is plotted in the bar charts. Exposure times were 250 ms for transferrin in the control samples and 50 ms in the Rab14- and FAM116A-depleted samples. This was not corrected for in the bar graph. Errors bars indicate the standard deviation (n = 5). (C) HeLa cells were treated with control, Rab4, Rab11, Rab14, and FAM116A siRNA duplexes for 72 hr and then serum starved for 16 hr to match the conditions used for transferrin uptake experiments. The cells were then lysed in SDS-PAGE sample buffer and western blotted as indicated in the figure. Tubulin was used as a loading control. See also Figure S3.

Assays measuring the transport of epidermal growth factor (EGF) and transferrin were performed to identify at which step in the endocytic pathway Rab14 and FAM116A function (Figure 4). These assays revealed that EGF uptake and transport were similar in all conditions (Figures 4A and 4B), suggesting that the trafficking pathway to early endosomes, late endosomes, and lysosomes is not perturbed in the absence of Rab14 or FAM116A. Inspection of the transferrin and transferrin receptor staining in Rab14- or FAM116A-depleted cells revealed a 5-fold higher signal at all time points. The images shown in the control and the Rab14- and FAM116A-depleted samples were captured using 250 and 50 ms exposure times, respectively (Figure 4A). Western blotting confirmed that this increase in signal was due to elevated levels of transferrin receptor and that the level of the lysosome or Golgi proteins, LAMP1 and GM130, was unchanged (Figure 4C). In addition, endocytosed transferrin accumulated in large punctate structures in the Rab14- and FAM116A-depleted cells (Figure 4A), and recycling was delayed relative to the control (Figure 4B). This was especially noticeable at 45–60 min, when most of the endocytosed transferrin had been lost from the control cells (Figure 4A). Live cell imaging of transferrin uptake confirmed that transferrin passed through a Rab14-positive compartment (Figure S3A) and that transferrin recycling was blocked in Rab14-depleted cells (Figure S3B and Movie S5).

**Figure 5:**
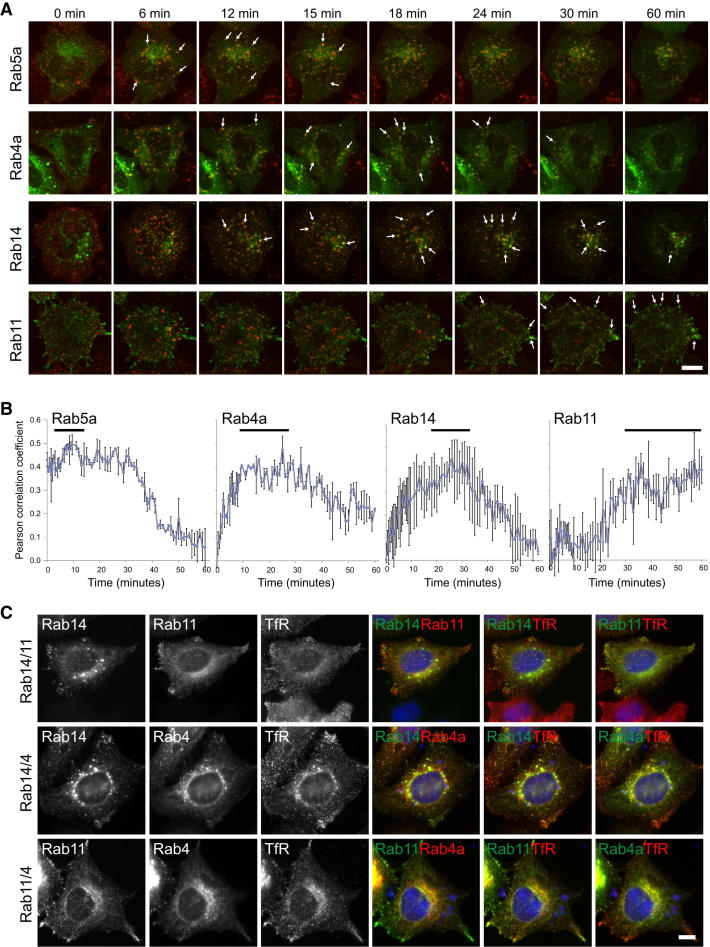
Rab14 Defines an Intermediate Endocytic Recycling Compartment (A) HeLa cells expressing eGFP-Rab GTPases as indicated were used for live cell imaging of transferrin uptake assays. Maximum intensity projection images of the entire cell volume are shown for the time points indicated. Arrows mark regions over colocalization. (B) Colocalization of the eGFP-Rab GTPases and transferrin were calculated using the volume quantitation tools of Volocity 5. Cell volumes were outlined and Pearson correlation coefficients for colocalization of the two markers calculated for each time point. Pearson correlation coefficients are plotted against time, and error bars indicate the standard error of the mean (n = 5). (C) HeLa cells coexpressing eGFP-Rab14 and either mCherry-Rab4 or Rab11 were fixed and stained with antibodies to the transferrin receptor. The scale bar indicates 10 μm in all panels.

Previous work has shown that Rab5, Rab4, and Rab11 define discrete membrane domains within the endocytic recycling pathway (Sönnichsen et al., 2000). To place these in order with respect to Rab14, live cell imaging of transferrin recycling in cells expressing the different Rabs was performed, and the colocalization of the transferrin and the different Rabs were measured over time (Figure 5). As expected, Rab5 and Rab11 defined early and late steps in the recycling pathway, and Rab4 was found on both early and later compartments (Figures 5A and 5B) as shown previously (Sönnichsen et al., 2000). Rab14 showed maximum colocalization with transferrin at intermediate time points from 20 to 30 min (Figures 5A and 5B), consistent with the time at which FAM116A and endocytosed transferrin overlap (Figure 3D). Direct comparison of Rab14 with Rab11 and Rab4 revealed that these three Rabs define different compartments (Figure 5C). Rab14 and its GEF FAM116A therefore define an intermediate step in the endocytic recycling pathway, after Rab5 and prior to the action of Rab11. The increased levels of transferrin receptor seen following Rab14 or FAM116A depletion indicate that these cells may be starved of iron and therefore upregulate the levels of the transferrin receptor to compensate. These findings suggest that an endocytic trafficking defect, specifically in the endocytic recycling pathway, underpins the cell migration defect of Rab14- and FAM116A-depleted A549 cells.

### N-Cadherin Accumulation at Cell-Cell Junctions in Rab14-Depleted Cells

**Figure 6:**
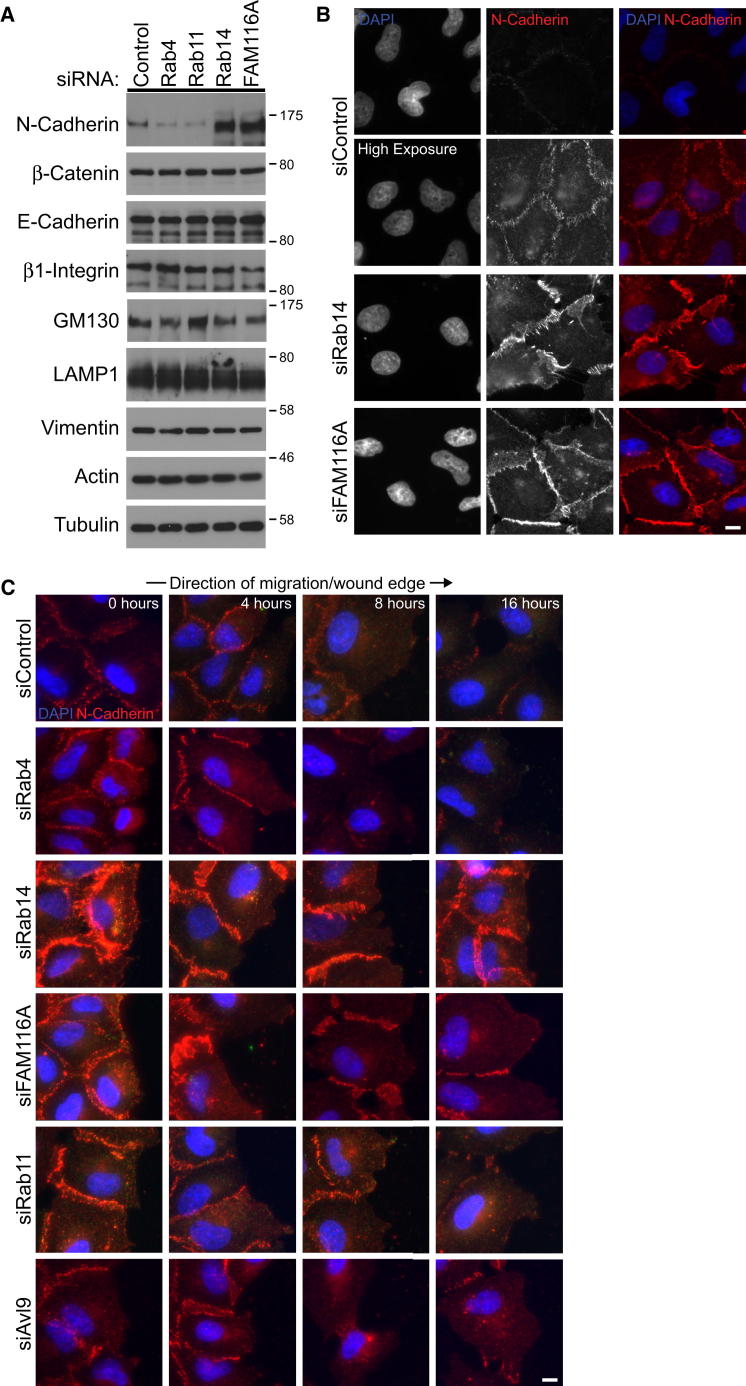
Abnormal Adherens Junctions in Rab14- and FAM116-Depleted Cells (A) A549 cells were treated with control, Rab4, Rab11, Rab14, FAM116A, and Avl9 siRNA duplexes for 72 hr. The cells were lysed in SDS-PAGE sample buffer and then western blotted as indicated in the figure. Tubulin was used as a loading control. (B) A549 cells were treated with control, Rab14, and FAM116A siRNA duplexes for 72 hr, fixed, and then stained with DAPI and antibodies to N-cadherin. (C) A549 cells were treated with control, Rab4, Rab11, Rab14, FAM116A, and Avl9 siRNA duplexes for 72 hr. The cell monolayers were then scratched, samples fixed at 0, 4, 8, and 16 hr, and then stained with DAPI and antibodies to N-cadherin. Images are oriented so the wound is to the right. Scale bar indicates 10 μm in all images. See also Figure S6.

Cell migration defects in previous studies have been linked to defective polarization of the cytoskeleton and Golgi apparatus. However, in Rab14- and FAM116A-depleted cells, polarization of the cytoskeleton and Golgi apparatus toward the wound edge appear normal (Figures S4A and S4B), eliminating this as the cause of the cell migration defect. Other studies have implicated the Rab11 subfamily GTPases Rab4, Rab11, and Rab25 in integrin and E-cadherin trafficking during cell migration in a number of in vitro and in vivo systems. Staining and western blot analysis of Rab14- or FAM116A-depleted cells did not show any defects in integrin levels or localization in A549 cells under the conditions used in this study but did reveal an increase in the amount of N-cadherin (Figure 6A; data not shown). Depletion of Rab4 or Rab11 did not result in altered N-cadherin levels (Figure 6A). The levels of E-cadherin, β-catenin, vimentin, and actin were similar to the control samples (Figure 6A), suggesting that the cells are not undergoing a transition from an epithelial to a mesenchymal state or vice versa. The localization of N-cadherin was then investigated. In control cells, N-cadherin levels were low, and a higher exposure was needed to visualize the cell-cell junction staining (Figure 6B). By contrast, a defined cell-cell junction staining of N-cadherin was visible even with a short exposure in the Rab14- and FAM116A-depleted cells (Figure 6B). This suggests that defective regulation of cell-cell adherens junctions may underlie the migration defect seen in these cells. To investigate this idea, scratch-wound migration assays were performed on cells depleted of Rab4, Rab11, Rab14, FAM116A, or Avl9. In the control cells or cells depleted of Rab4, Rab11, or Avl9 the cell-cell junction associated staining of N-cadherin was reduced or lost after 4–8 hr when the cells separate from the edge of the cell sheet and migrate, or attempt to migrate, into the free space created by the wound (Figure 6C). Rab14- and FAM116A-depleted cells fail to separate from the edge of the wound and retain defined staining of N-cadherin at cell-cell junctions after 16 hr (Figure 6C).

### N-Cadherin Silencing Rescues the Rab14 and FAM116 Migration Defect

**Figure 7:**
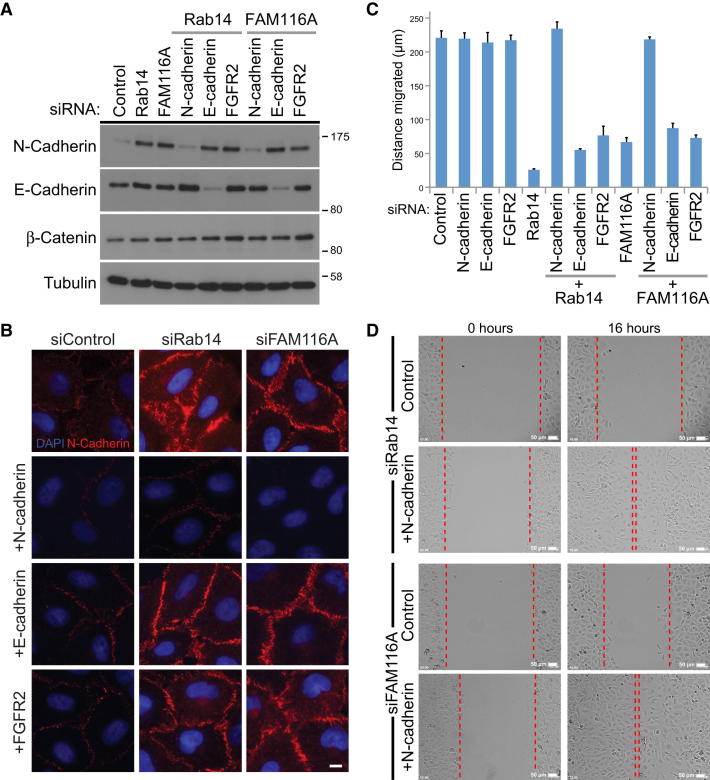
N-Cadherin Depletion Rescues the Migration Defect in Rab14- and FAM116-Depleted Cells (A and B) A549 cells were treated with control, Rab14, FAM116A, N-cadherin, E-cadherin, and FGFR2 siRNA duplexes alone or in the combinations indicated for 72 hr (A). The cells were then western blotted for N-cadherin, E-cadherin, or tubulin as a loading control or (B) fixed and then stained with DAPI and antibodies to N-cadherin. Scale bar indicates 10 μm. (C) A549 cells were treated with control, Rab14, FAM116A, N-cadherin, E-cadherin, and FGFR2 siRNA duplexes alone or in the combinations indicated for 72 hr. The cell monolayers were then scratched and imaged for 16 hr. Cell migration was measured and is plotted on the graph with error bars to show the standard error of the mean (n = 3). (D) Images are shown from the 0 and 16 hr time points for the Rab14- and FAM116A-depleted cells in the presence and absence of N-cadherin siRNA. Scale bar indicates 50 μm. See also Figure S5.

These findings support the idea that in the absence of the Rab14 and FAM116 recycling pathway, cells are unable to remodel cell-cell adherens junctions linking cells at the wound edge with the adjacent cell-sheet. Adherens junctions were therefore disrupted in two different ways: by specific silencing of cadherins or calcium chelation. Cells were simultaneously depleted of Rab14 or the Rab14 GEF FAM116A and the N- and E-cadherin adherens junction proteins (Figure 7A). Since the fibroblast growth factor receptor (FGFR2) has been linked to N-cadherin function in some cell migration events, this was also targeted (Cavallaro and Dejana, 2011; Cavallaro et al., 2001; Hulit et al., 2007; Suyama et al., 2002; Williams et al., 2001). Depletion of Rab14 or FAM116A resulted in increased levels of N-cadherin at cell-cell junctions, and this was reversed by knockdown of N-cadherin but not E-cadherin or FGFR2 (Figure 7B). Consistent with the hypothesis proposed above, N-cadherin depletion also rescued the cell migration defect seen in Rab14- and FAM116A-depleted cells (Figures 7C and 7D). Depletion of E-cadherin or FGFR2 had no obvious effect on migration (Figures 7C and 7D). Since cell-cell junctions require the presence of extracellular calcium, a simple way to disrupt them is with calcium chelation. The calcium-chelating agent EDTA was therefore titrated into cell migration assays to define a concentration that would not prevent cell adhesion to the dish surface or inhibit migration of control cells. Above 1 mM EDTA, A549 cell migration and adhesion were reduced (Figure S5A), and this concentration was therefore used for further experiments. Addition of 1 mM EDTA to Rab14- or FAM116A-depleted cells suppressed the cell migration defect (Figures S5B and S5C). By contrast, the cell migration defects seen with Rab4, Rab11, or Avl9 were not altered by calcium chelation (Figures S5B and S5C). Following EDTA treatment, N-cadherin staining at the cell-cell junctions in Rab14- and FAM116A-depleted cells at the wound edge was rapidly lost (Figure S5D), correlating with the increased migration (Figures S5B and S5C). Together, these results show that the elevated levels of N-cadherin at the cell-surface and increased cell-cell junction formation are the primary cause of the migration defect in cells lacking Rab14 and FAM116 function.

### N-Cadherin Shedding by ADAM9/10 Is Regulated by Rab14 and FAM116A

**Figure 8:**
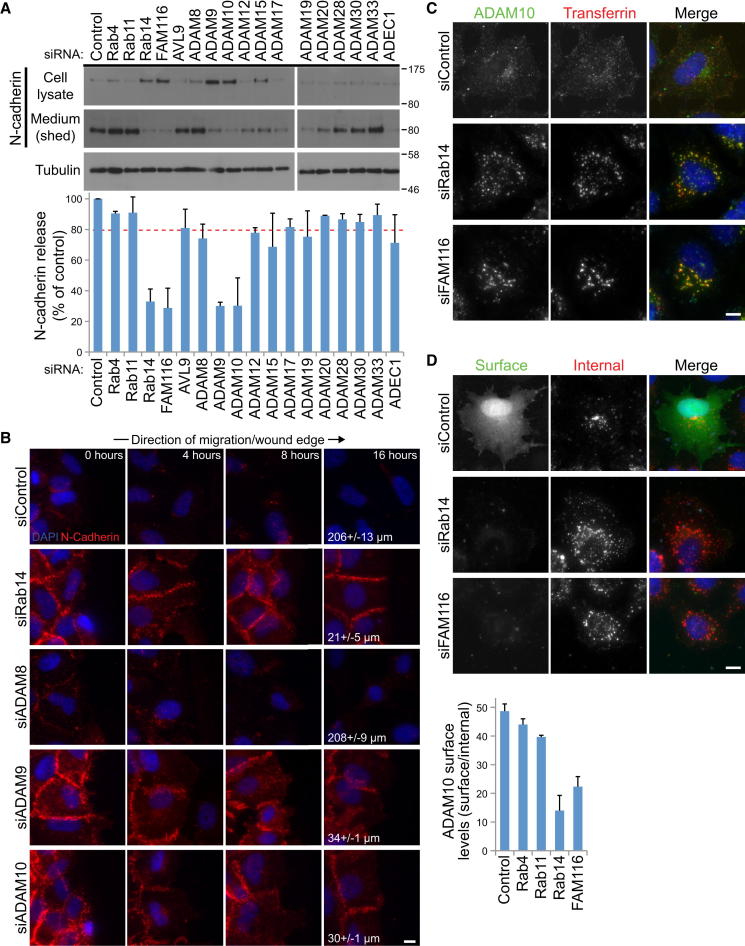
Rab14 and FAM116 Regulate ADAM10 Localization and N-Cadherin Shedding (A) A549 cells were treated with Rab, Rab GEF, and ADAM protease-specific siRNA duplexes as indicated for 72 hr. After 48 hr, the cells were washed three times in serum-free medium and left in serum-free medium for 24 hr. Proteins in the medium were then recovered by TCA precipitation, and equivalent aliquots of the cell lysate and medium were western blotted for N-cadherin or tubulin as a loading control. The amount of shed N-cadherin was calculated for two independent experiments and is plotted in the bar graph. (B) A549 cells were treated with control, Rab14, ADAM8, ADAM9, and ADAM10 siRNA duplexes for 72 hr. The cell monolayers were then scratched, samples fixed at 0, 4, 8, and 16 hr, and then stained with DAPI and antibodies to N-cadherin. Images are oriented so the wound is to the right. Scale bar indicates 10 μm in all images. The distance migrated is shown with a standard deviation (n = 3). (C) Cells treated as above were used for transferrin uptake assays and then after 30 min were fixed and stained with ADAM10 antibodies. (D) ADAM10 antibodies were bound to the cell-surface on ice for 60 min; the cells were then fixed and stained with secondary antibodies to detect cell surface ADAM10 (green). The cells were then postfixed, permeabilized, and stained using a standard protocol to detect the internal pool of ADAM10 (red). The cell surfaces levels of ADAM10 were measured as the ratio of surface/internal ADAM10 staining. Error bars indicate the standard deviation (n = 3). The scale bar marks 10 μm in all panels.

Like numerous other cell-surface membrane proteins, the levels of cadherins are regulated by transmembrane proteases of the disintegrin and metalloprotease domain (ADAM) family. ADAMs cleave the extracellular domain of the target, resulting in its release from the membrane, a process often termed shedding. Increased levels of N-cadherin at the cell surface could therefore be due to altered recycling of an ADAM family protease. To test this idea, cells were depleted of Rabs and Rab GEFs required for cell migration, and samples of the cells and growth media were analyzed by western blotting (Figure 8A). This showed that N-cadherin shedding was reduced in cells lacking Rab14 or its GEF FAM116 but was not altered in Rab4-, Rab11-, or Avl9-depleted cells (Figure 8A). A library of the catalytically active ADAM proteases was then screened to identify which family members were required for N-cadherin shedding and cell migration. Cells depleted of ADAM9 or ADAM10/Kuzbanian but not of other ADAM family proteases showed reduced levels of N-cadherin shedding (Figure 8A) and increased cell-surface staining of N-cadherin (Figure 8B). They also displayed a migration defect similar to that of Rab14- or FAM116-depleted cells and retained N-cadherin at cell-cell junctions after 16 hr (Figure 8B). Cells depleted of ADAM8 showed cell surface levels of N-cadherin and migration behavior similar to the control (Figure 8B). These data suggest that altered trafficking of ADAM10 to the cell surface may explain the migration defect and altered N-cadherin levels in Rab14- or FAM116-depleted A549 cells. Supporting this idea, ADAM10 accumulated in an intracellular transferrin-positive compartment upon depletion of Rab14 or FAM116 (Figure 8C), and cell-surface levels of ADAM10 were reduced in cells depleted of Rab14 or FAM116 but not of Rab4 or Rab11 (Figure 8D).

Taken together, the findings in this study show that Rab14 and its GEF FAM116A are components of the endocytic recycling pathway. Furthermore, they reveal that Rab14 and FAM116A define a trafficking route important for regulating N-cadherin by ADAM family proteases and therefore for the control of cell-cell adherens junctions.

## Discussion

### Differential N- and E-Cadherin Processing

Rab11-related GTPases, Rab4, Rab11, and Rab25, have well-characterized roles in integrin and E-cadherin trafficking through recycling endosomes during cell migration and epithelial polarization events (Baum and Georgiou, 2011; Caswell et al., 2009). The results presented here show that Rab14, the final member of this family, also plays a role in cell migration events by regulating the turnover of N-cadherin at the cell surface. This increase in N-cadherin levels is due to altered recycling of ADAM family transmembrane proteases, specifically ADAM9 and ADAM10. ADAM10 has previously been shown to directly control the cleavage of cadherins and thereby regulate their levels at the cell surface (Maretzky et al., 2005; Reiss et al., 2005). Since ADAM10 accumulates in a transferrin-positive compartment in cells depleted of Rab14 or FAM116 and is therefore reduced at the cell surface, this results in less N-cadherin shedding and increased cell-associated N-cadherin. Interestingly, this may also explain why levels of the transferrin receptor are increased, because ADAMs have also been implicated in shedding of this receptor (Bech-Serra et al., 2006). Other studies have shown that shedding of some ligands, such as collagen XVII, depends on both ADAM9 and ADAM10 (Franzke et al., 2009) and that ADAM9 may act by processing ADAM10 (Tousseyn et al., 2009).

Exactly how N- and E-cadherins would be discriminated by such a mechanism is not clear, and further regulation inputs must therefore be required. Interestingly, recent evidence shows that E-cadherin interacts with ephrin B receptors and modulates its processing by ADAM10 (Solanas et al., 2011), and it is possible that mechanisms like this enable independent regulation of the different cadherin subtypes. N-cadherin has been shown to directly interact with the fibroblast growth factor (FGF) receptor and to be involved in FGF-receptor signaling during some cell migration events and in metastasis (Cavallaro and Dejana, 2011; Cavallaro et al., 2001; Hulit et al., 2007; Suyama et al., 2002; Williams et al., 2001). Intriguingly, Rab14 has been previously shown to function in the trafficking of the fibroblast growth factor receptor 2 (FGFR2) during early embryonic development (Ueno et al., 2011). However, under the conditions used here, FGFR2 does not appear to be crucial for explaining the migration defect in Rab14- and FAM116A-depleted A549 cells, suggesting that it does not play a role in N-cadherin regulation.

### Multiple Rabs Defining an Endocytic Recycling Network

Cells independently regulate the cell-surface recycling of many different classes of membrane protein to different domains of the plasma membrane. To date, much of the focus has been on the role of Rab11 in endocytic recycling. However, Rab11 function alone cannot explain the behavior of all recycling molecules. This is particularly obvious in polarized epithelial cells, where Rab11 is required for recycling to the basolateral surface only (Tzaban et al., 2009). By contrast, other evidence suggests that Rab14 may be required for transport to the apical surface (Kitt et al., 2008), fitting with the idea that Rab11 and Rab14 control the recycling to different domains of the cell surface. The results presented here and elsewhere also suggest that Rab11 and Rab14 also regulate the trafficking of different cargo. These findings and the observed behavior of different recycling cargo are difficult to explain in terms of a simple linear transport pathway. In fact, the apical and basolateral endocytic recycling pathways in polarized cells form an intersecting network (Gibson et al., 1998; Odorizzi et al., 1996). The Rab-dependency of recycling will therefore be determined by the way in which a particular cargo molecule traverses this network and may not conform to a model invoking simple linear series of membrane compartments.

### Rab GEF Families

Previous studies have shown that the core DENN family of proteins have specific Rab substrates (Allaire et al., 2010; Sato et al., 2008; Yoshimura et al., 2010) and explained the structural basis for this Rab GEF activity (Wu et al., 2011). The best understood of these is the DENND1 subfamily, which has been shown to control Rab35 in an endocytic pathway required for yolk protein receptor recycling in *C. elegans*, MHC-I recycling in mammals, and the uptake of the causative agent of shigellosis – Shiga toxin (Allaire et al., 2010; Sato et al., 2008; Yoshimura et al., 2010). The results presented here extend the known Rab GEFs by adding the DENN-related proteins to list of proteins with biochemically defined Rab GEF activity. Because of their similarity with the core DENN family, FAM116A and FAM116B have been referred to as DENND6A and B (Marat et al., 2011), and this seems reasonable, given the fact that they share not only homology but also Rab GEF activity.

Whether the remaining DENN-related proteins, Avl9, FAM45A, and KIAA1147, are Rab GEFs remains unclear. Avl9 was first described in budding yeast as a secretion mutant, showing synthetic lethality with the dynamin Vps1 and the Apl2 subunit of the clathrin adaptor complex 1 (Harsay and Schekman, 2007). Subsequent work suggested that Gtr2, a Ras superfamily GTPase outside the Rab subfamily, was the target of Avl9 (Zhang et al., 2010). However, direct biochemical evidence for GEF activity was lacking in these studies. The similarity of the mammalian Avl9 and Rab4 and Rab11 migration defects suggested that Avl9 might be a Rab11 or Rab4 GEF. However, despite extensive efforts, it was not possible to demonstrate GEF activity toward either Rab4 or Rab11. One possibility is that Avl9, like Rab GEFs of the Mon1-Ccz1 and Ric1-Rgp1 families, requires an additional subunit for activity (Barr and Lambright, 2010). The question of whether or not Avl9 is a Rab GEF therefore remains unresolved, and extensive further studies will therefore be required. FAM45A and KIAA1147 depletion did not give rise to migration defects, so these do not appear to be Rab4 or Rab11 GEFs and might not even be Rab GEFs. A number of Rabs, including Rab2 found on the Golgi apparatus and secretory granules, Rab18 found on lipid droplets, and Rab30, Rab33A, Rab33B, and Rab43 linked to Golgi apparatus function also lack any known GEF activity. Therefore, even with recent advances in our understanding of Rab activation and the function of Rab GEFs, further work is required to create a complete picture of Rab regulators and their cellular functions. By describing a GEF regulator for Rab14 and explaining its cellular function, this study is an important step toward this goal.

## Experimental Procedures

### Reagents and Antibodies

General laboratory chemicals were obtained from Sigma-Aldrich (St. Louis, MO, USA) and Fisher Scientific (Hampton, NH, USA). Antibodies were raised against full-length recombinant hexahistidine-tagged Rab14 and affinity purified on the antigen coupled to Affigel-15 (Bio-Rad, Hercules, CA, USA). Commercially available antibodies were used to α-tubulin (mouse DM1A; Sigma-Aldrich); EEA1 (rabbit #2411; Cell Signaling, Danvers, MA, USA); GM130 (mouse clone 35; BD Biosciences, Franklin, NJ, USA), LAMP1 (mouse 1D4B; Developmental Studies Hybridoma Bank, University of Iowa, Iowa City, IA, USA); TGN46 (sheep AHP500; Serotec, Raleigh, NC, USA), TfR (rabbit CBL47; Millipore, Billerica, MA, USA); CI-MPR (2G11; Abcam, Cambridge, UK); Pericentrin (rabbit AB4448; Abcam); E-cadherin (rabbit monoclonal CDH1; Epitomics, Burlingame, CA, USA); N-cadherin (sheep AF6426; R&D Systems, Minneapolis, MN, USA), β-catenin (rabbit #9581, Cell Signaling); β1-integrin (mouse MCA2028, Serotec; mouse 9EG7, BD Biosciences; rat MAB1997, Millipore). Secondary antibodies raised in donkey to mouse, rabbit, sheep/goat, and human conjugated to HRP, Alexa-488, Alexa-555, Alexa-568, and Alexa-647 were obtained from Molecular Probes (Eugene, OR, USA) and Jackson ImmunoResearch Labs (West Grove, PA, USA).

### Molecular Biology

The libraries of Rab GTPases and human DENN coding sequences have been described previously (Yoshimura et al., 2007, 2010). Mutagenesis was performed using the QuikChange method in accordance with the protocol (Stratagene, La Jolla, CA, USA). Duplexes for siRNA were obtained from QIAGEN (Venlo, The Netherlands) or Dharmacon (Lafayette, CO, USA) and are listed in Table S1. A GL2 duplex was used as a control in all siRNA experiments. Mammalian expression constructs were made using pcDNA4/TO and pcDNA5/FRT/TO vectors (Invitrogen). Bacterial expression constructs were made using pQE32 (QIAGEN), pMal (New England Biolabs, Ipswich, MA, USA), and pFAT2, encoding the Hexahistidine-tag, Hexahistidine-maltose-binding protein, and Hexahistidine-glutathione-S-transferase, respectively.

### Cell Culture and Protein Purification

A549, HeLa, and HEK293 cells were cultured in Dulbecco's modified Eagle's medium (DMEM) containing 10% bovine calf serum (Invitrogen, Carlsbad, CA, USA) at 37°C and 5% CO_2_. For plasmid transfection and siRNA transfection, Mirus LT1 (Mirus Bio LLC, Madison, WI, USA) and Oligofectamine (Invitrogen), respectively, were used in accordance with the manufacturer's instructions. Proteins were purified and expressed in bacteria or mammalian cells as described previously. Purified proteins were dialysed against Tris-buffered saline (50 mM Tris-HCl [pH 7.4], 150 mM NaCl) and then snap frozen in liquid nitrogen for storage at −80°C.

### Nucleotide Binding and GEF Assays

Nucleotide loading was carried out as follows: 10 μg GST-tagged Rab was incubated in 50 mM HEPES-NaOH (pH 6.8), 0.1 mg/ml BSA, 125 μM EDTA, 10 μM Mg-GDP, and 5 μCi [^3^H]-GDP (10 mCi/ml; 5000 Ci/mmol) in a total volume of 200 μl for 12 hr at 4°C. For standard GDP-releasing GEF assays, 100 μl of the loading reaction was mixed with 10 μl 10 mM Mg-GTP, 10-100 nM GEF protein to be tested or a buffer control, and adjusted to 120 μl final volume with assay buffer. The GEF reaction occurred for 20 min at 30°C. After this, 2.5 μl were taken for a specific activity measurement; the remainder was split into two tubes and then incubated with 500 μl ice-cold assay buffer containing 1 mM MgCl_2_ and 20 μl packed glutathione-sepharose for 60 min at 4°C. After washing three times with 500 μl ice-cold assay buffer, the sepharose was transferred to a vial containing 4 ml scintillation fluid and counted. The amount of nucleotide exchange was calculated in pmoles GDP-released. For GTP-binding assays, the following modifications were made: only unlabelled GDP was used in the loading reaction; in the GEF reaction, 0.5 μl 10 mM GTP and 1 μCi [^35^S]-GTPγS (10 mCi/ml; 5000 Ci/mmol) were used. The amount of nucleotide exchange was calculated in pmoles GTP-bound.

### Cell Migration Assays

For live cell imaging, 10,000 A549 cells per well were plated in 24-well plastic dishes. After 24 hr, cells were treated with siRNA duplexes then left for a further 72 hr. Wounds were scratched in the monolayer using a 200 μl pipette tip (Diamond Standard Tip, Gilson Inc., Middletown, WI, USA), and the cells then left to recover for 30 min at 37°C. All timings start after this recovery period. Imaging was then performed at 37°C in CO_2_ independent growth medium (Invitrogen) using an Olympus IX81-ZDC inverted microscope with a 10× 0.6NA air objective, a CoolSNAP HQ2 camera (Roper Scientific, Trenton, NJ, USA), and an XY motorised piezo Z-stage (Applied Scientific Instrumentation) under the control of Metamorph 7.5 software (Photometrics UK Ltd., London, UK). Brightfield images were collected using 10 ms exposures every 5 min for 16 hr. Images were placed into Adobe Illustrator CS3 to produce the figures. For fixed-cell assays, A549 cells were plated on coverslips in 12-well plates and then treated in the same way as live cell assays. At the time points indicated in the figures, the cells were fixed and then processed for immune fluorescence microscopy.

### Transferrin and EGF Uptake Assays

EGF and transferrin (Tf) coupled to Alexa Fluor 488 or Alex Fluor 555 (40× stock, 200 μg/ml) (Molecular Probes/Invitrogen) were stored as stock solutions in PBS at −20°C. For uptake assays, HeLa cells plated on glass coverslips at a density of 70,000 cells/well of a 6-well plate were washed three times with serum-free growth medium 36 hr after plating and then incubated in serum-free growth medium for 15 to 16 hr at 37°C and 5% CO_2_. Coverslips were then washed three times in ice-cold PBS and placed on 40 μl drops of uptake medium (DMEM, 2% [wt/vol] bovine serum albumin, 20 mM HEPES-NaOH [pH 7.5]) containing 5 μg/ml EGF and Tf on an ice-cold metal plate covered in Parafilm (Pechiney Plastic Packaging, Menasha, WI, USA). For some experiments, cells were transfected with eGFP-Rab constructs for 18 hr in advance. After 30 min incubation, the coverslips were washed three times in ice-cold PBS to remove excess ligand. One coverslip was fixed to give the total bound ligand, while the remaining coverslips were transferred to a 6-well plate containing prewarmed growth medium and incubated at 37°C and 5% CO_2_. At the time points indicated in the figures, the cells were fixed and then processed for immune fluorescence microscopy. For live cell imaging, cells were plated in 2 cm dishes with a coverglass window in the bottom. For EGF and Tf binding, a 60 μl drop of uptake medium was placed on top of the coverglass window. Imaging was performed at 37°C in 5% CO_2_ using the 60×1.42 NA oil immersion objective of an Ultraview Vox spinning disk confocal system (Perkin Elmer, Waltham, MA, USA). Image stacks of 25–35 planes spaced 0.5–0.7 μm were taken every minute for 2 hr. Exposure times were 10–33 ms at 3% laser power for both the 488 and 555 nm probes. Maximum intensity projection images of the fluorescent channels were cropped in NIH ImageJ and placed into Adobe Illustrator CS3 to produce the figures. Intensity and colocalization measurements were performed on the full 3D data set using the volume quantitation and object tracking tools of Volocity 5 (Perkin Elmer).

### Fixed-Cell Microscopy

Fixed samples on glass slides were imaged using a 60× 1.35 NA oil immersion objective on a standard upright microscope equipped with a CoolSNAP HQ2 camera (Roper Scientific) under the control of Metamorph 7.5 software. Images were cropped in Adobe Photoshop CS3 or ImageJ and placed into Illustrator CS3 without performing any other contrast adjustments or image manipulations to produce the figures.

## Supplemental Information

Document S1. Figures S1–S5

Table S1. Sequences of All the siRNA Duplexes Used in the Study, Related to Figures 1 and 2

Movie S1. Rab GTPases Giving Rise to Migration Defects Movies showing the control, Rab4-, Rab11-, and Rab14-depleted cells from the migration screen described in Figure 1. Images were taken every 5 min for 16 hr. The position of the wound edges at t0 and 16 hr are shown. Migration was calculated from the difference in these values.

Movie S2. Migration of Rab14-Depleted Cells Enlarged movies showing the control and Rab14-depleted cells from the migration screen described in Figure 1. Images were taken every 5 min for 16 hr. The position of the wound edge at t0 is shown.

Movie S3. Candidate Rab GEFs Required for Cell Migration Movies showing the control, Avl9, and FAM116A-depleted cells from the migration screen described in Figure 2. Images were taken every 5 min for 16 hr. The position of the wound edges at t0 and 16 hr are shown. Migration was calculated from the difference in these values. The enlarged movies show more detail of the events at the wound edge.

Movie S4. Transferrin Recycles through a FAM116A-Positive Compartment Transferrin (red) uptake was imaged every minute in HeLa cells expressing eGFP-FAM116A (green), as described in Figure 4B. Transient overlap (yellow) between the transferrin and eGFP-FAM116 can be seen prior to loss of the transferrin signal, when the receptor is recycled to the cell surface.

Movie S5. Transferrin Recycling in Control and Rab14-Depleted Cells Transferrin uptake (red) was imaged every minute in control and Rab14-depleted cells. Transferrin is transported into the punctate structures and then recycled and lost in control cells. In Rab14-depleted cells, transferrin accumulates in large punctate structures.

Movie S6. Calcium Chelation Recovers the Migration Defect in Rab14-Depleted Cells Enlarged movies showing the control and Rab14-depleted cells in the presence or absence of 1 mM EDTA treatment, as described in Figures S6 and S7. Images were taken every 5 min for 16 hr.
