# Supplementary material for: Rab14 and Its Exchange Factor FAM116 Link Endocytic Recycling and Adherens Junction Stability in Migrating Cells
Source: Dev Cell. 2012 May 15;22-540(5):952–66. doi: 10.1016/j.devcel.2012.04.010 (PMC3383995; doi:10.1016/j.devcel.2012.04.010)
Supplement: Document S1. Figures S1–S5 [file mmc1.pdf]

Developmental Cell, Volume *22*

Supplemental Information

Rab14 and Its Exchange Factor FAM116 Link Endocytic Recycling and Adherens Junction Stability in Migrating Cells

Andrea Linford, Shin-ichiro Yoshimura, Ricardo Nunes Bastos, Lars Langemeyer, Andreas Gerondopoulos, Daniel J. Rigden, and Francis A. Barr

Supplemental Inventory

Figure S1 outlines the entire family of DENN and DENN-related domain containing putative Rab GEFs used in the screen shown in Figure 2.

Figure S2 complements Figure 3, and shows that FAM116 and Rab14 are present on a transferrin receptor positive compartment of the endocytic recycling pathway.

Figure S3 provides additional support for the data in Figure 4 showing that transferrin recycles through Rab14 positive compartment and that transferrin recycling is blocked in Rab14 depleted cells.

Figure S4 shows that reorientation of the Golgi and microtubule organising centre are not defective when Rab14 and FAM116 depleted cells are wounded under the conditions used in Figure 6.

Figure S5 is a supplement to Figure 7. It shows that using limited calcium chelation to disrupt adherens junctions can rescue the migration defect of Rab14 and FAM116 depleted cells.

Table S1 contains the sequences of all the siRNA duplexes used in the study.

Figure S1. Linford et al.

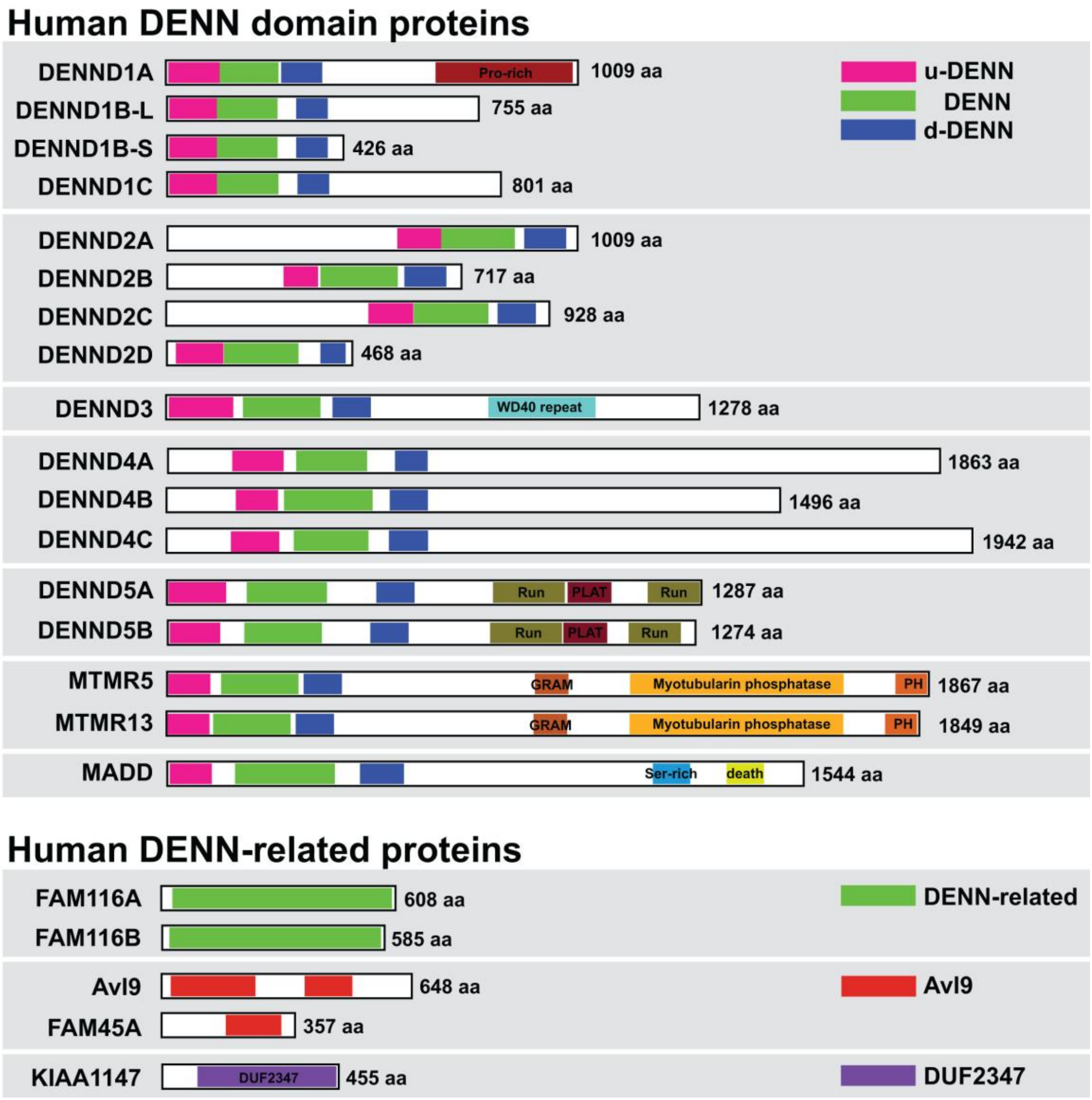

Figure S1. Summary of the DENN and DENN-related domain protein families. The human DENN domain and DENN-related proteins are shown in schematic form in the figure. Standard gene names are used.

Figure S2. Linford et al.

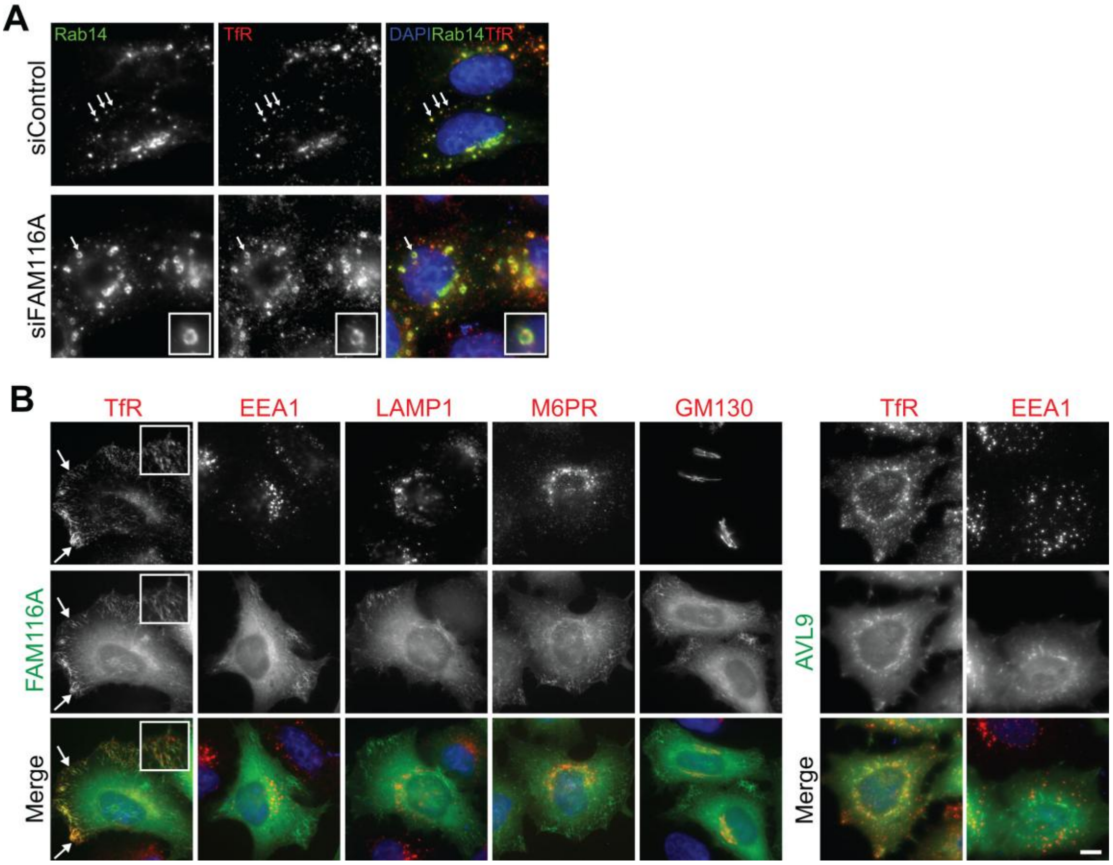

Figure S2. Transferrin recycles through a FAM116A positive compartment. (A) HeLa cells expressing eGFP-Rab14 were treated with control or FAM116A siRNA duplexes for 72 hours, fixed, and then stained for the transferrin receptor and DAPI. The enlarged area shows the overlap between Rab14 and transferrin receptor in FAM116A depleted cells. (B) HeLa cells expressing eGFP-FAM116A or eGFP-Avl9 were fixed, and then stained with antibodies to transferrin receptor (TfR; recycling endosomes), early-endosomal antigen 1 (EEA1; early endosomes), lysosomal acidic membrane protein 1 (LAMP1; lysosomes), mannose-6-phosphate receptor (M6PR; trans-Golgi and late endosomes), and the 130 kDa Golgi matrix protein (GM130; Golgi apparatus). Scale bars indicate 10 µm in all panels.

Figure S3. Linford et al.

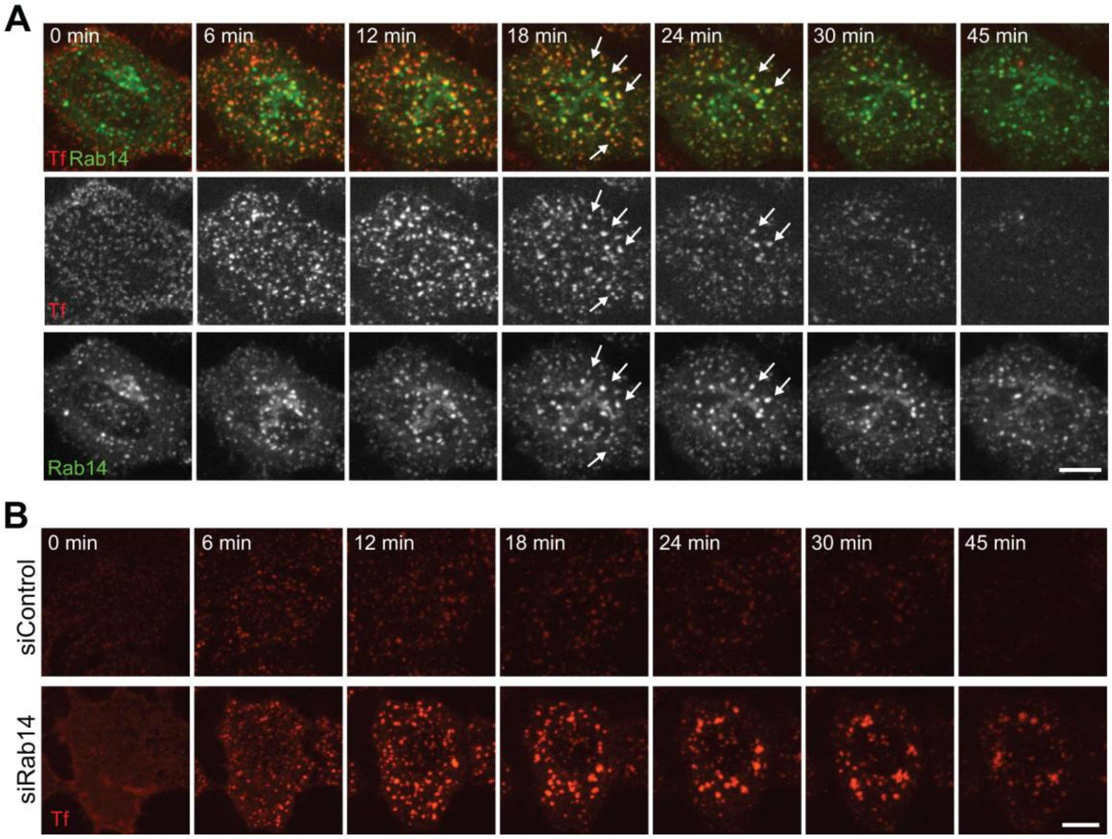

Figure S3. Transferrin recycles through a Rab14 positive compartment.

(A) Transferrin uptake assays were performed using HeLa cells transfected with eGFP-Rab14. Tf and eGFP-Rab14 were imaged every minute at 37°C using a spinning disk confocal microscope. (B) Transferrin uptake assays were performed using HeLa cells treated with control or Rab14 siRNA duplexes for 72 hours. Tf was imaged every minute at 37°C using a spinning disk confocal microscope. Scale bars indicate 10 µm in all panels.

Figure S4. Linford et al.

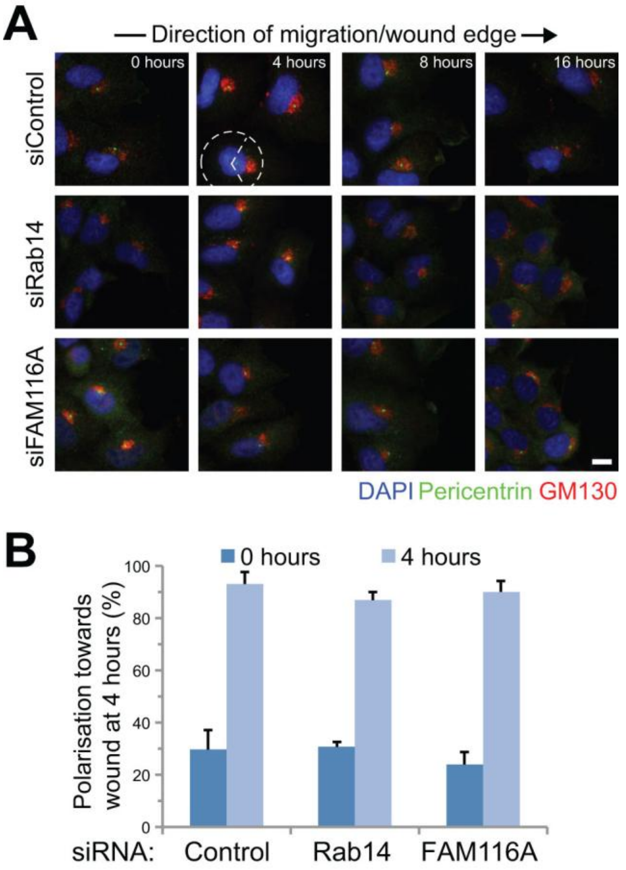

Figure S4. Analysis of Golgi and centrosome polarisation.

(A) A549 cells treated with control, Rab14, or FAM116A siRNA for 72 hours were scratch wounded and then left for 0, 4, 8, and 16 hours as indicated. The cells were fixed, and then stained with antibodies to the Golgi marker GM130, and the centriolar marker pericentrin. DNA was stained with DAPI. The scale bar is 10 µm. Cell polarisation towards the wound was assessed by drawing a circle centred over the nucleus, with a 120° segment facing the wound. Cells were polarised towards the wound, if the Golgi and centrosome were located in this segment. Images are oriented so the wound is to the right. (B) Cell polarisation at 0 and 4 hours is plotted in the bar graph. Bars indicate the standard error from the mean (n=3).

Figure S5. Linford et al.

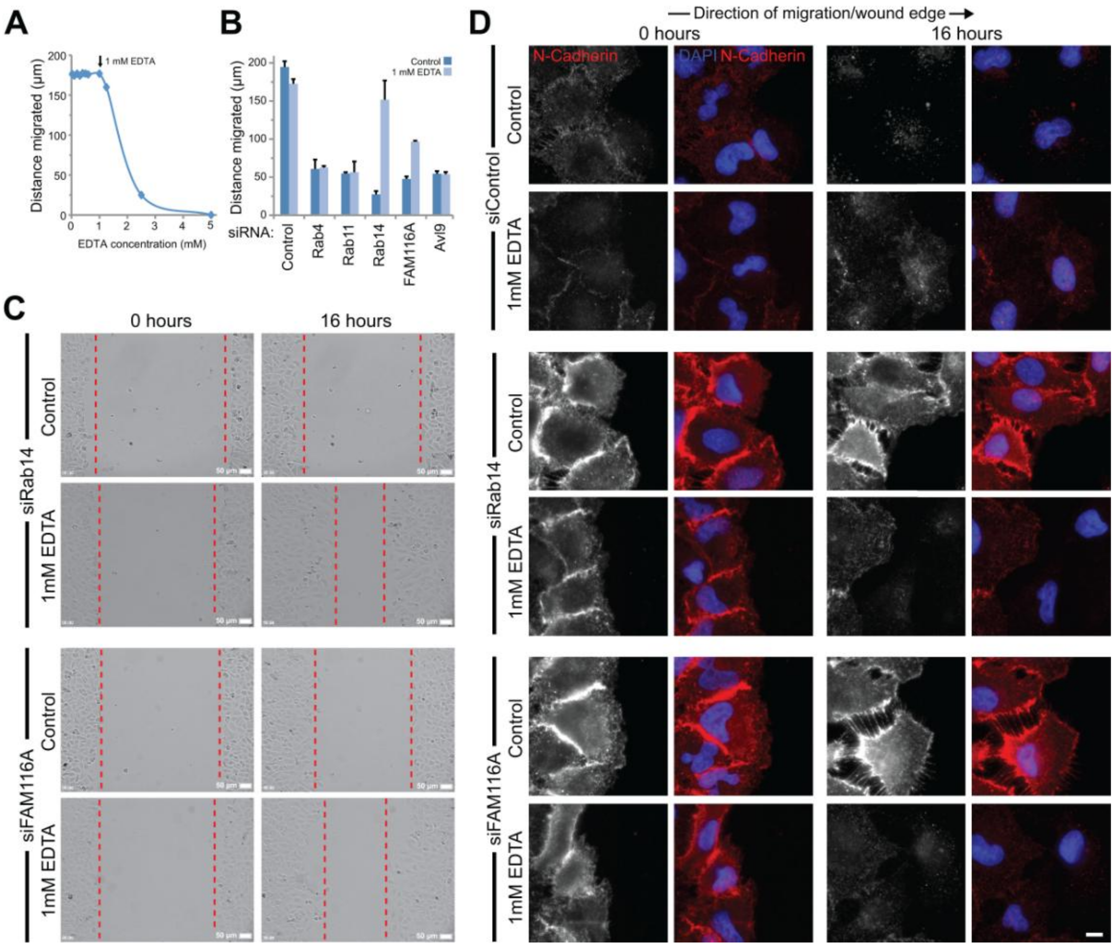

Figure S5. Reduced cell surface N-cadherin in Rab14 and FAM116 depleted cells following calcium chelation.

(A) EDTA was added to confluent monolayers of A549 cells to a final concentration of 25, 50, 100, 200, 300, 400, 500, 600 µM, or 1, 1.25, 2.5, 5 mM. The cells were then scratched to create a wound, left for 30 minutes to recover, and then imaged for 16 hours. Cell migration was measured and is plotted on the graph as a function of EDTA concentration. At 2.5 and 5 mM EDTA cell numbers were reduced due to adhesion defects. Error bars show the standard deviation from the mean (n=3). (B) A549 cells were treated with control, Rab14, or FAM116A siRNA duplexes for 72 hours. The cell monolayers were scratched, samples fixed at 0, 4, 8, and 16 hours, and then

stained with DAPI and antibodies to N-cadherin. Images are oriented so the wound is to the right. Images from the 0 and 16 hour time points are shown. (C) Images are shown from the 0 and 16 hours time points for the Rab14 and FAM116A depleted cells in the presence and absence of EDTA. Scale bar indicates 50 µm. (D) A549 cells were treated with control, Rab4, Rab11, Rab14, FAM116A, and Avl9 siRNA duplexes for 72 hours. The cell monolayers were then scratched and imaged for 16 hours in the presence or absence (control) of 1 mM EDTA. Scale bar indicates 10 µm.

Table S1.

(see accompanying Excel file)
