# Supplementary figures and images for: Rab14 and Its Exchange Factor FAM116 Link Endocytic Recycling and Adherens Junction Stability in Migrating Cells
Source: Dev Cell. 2012 May 15;22-540(5):952–66. doi: 10.1016/j.devcel.2012.04.010 (PMC3383995; doi:10.1016/j.devcel.2012.04.010)

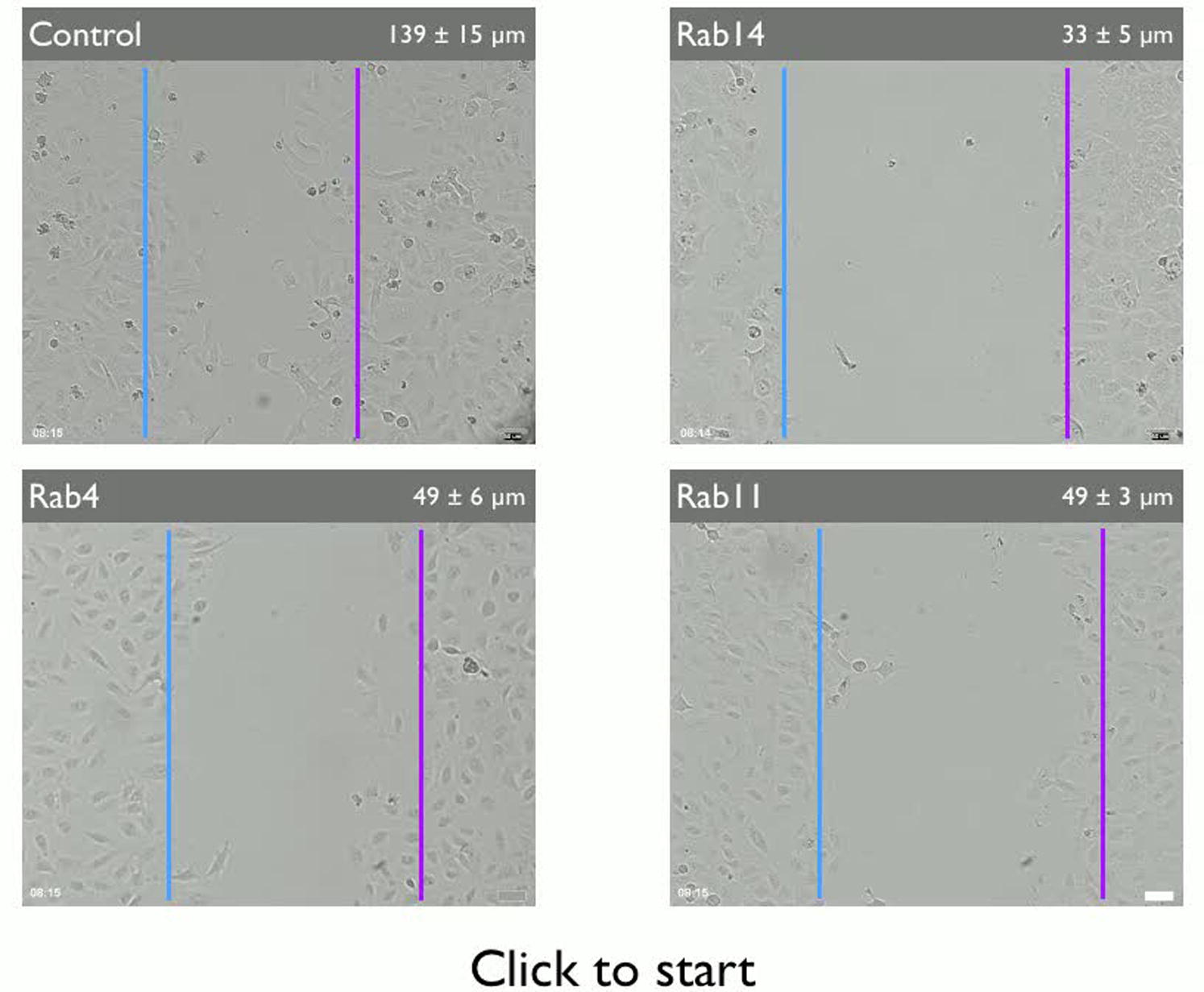

Supplement: Movie S1. Rab GTPases Giving Rise to Migration Defects — Movies showing the control, Rab4-, Rab11-, and Rab14-depleted cells from the migration screen described in Figure 1. Images were taken every 5 min for 16 hr. The position of the wound edges at t0 and 16 hr are shown. Migration was calculated from the difference in these values. [file mmc3.jpg]

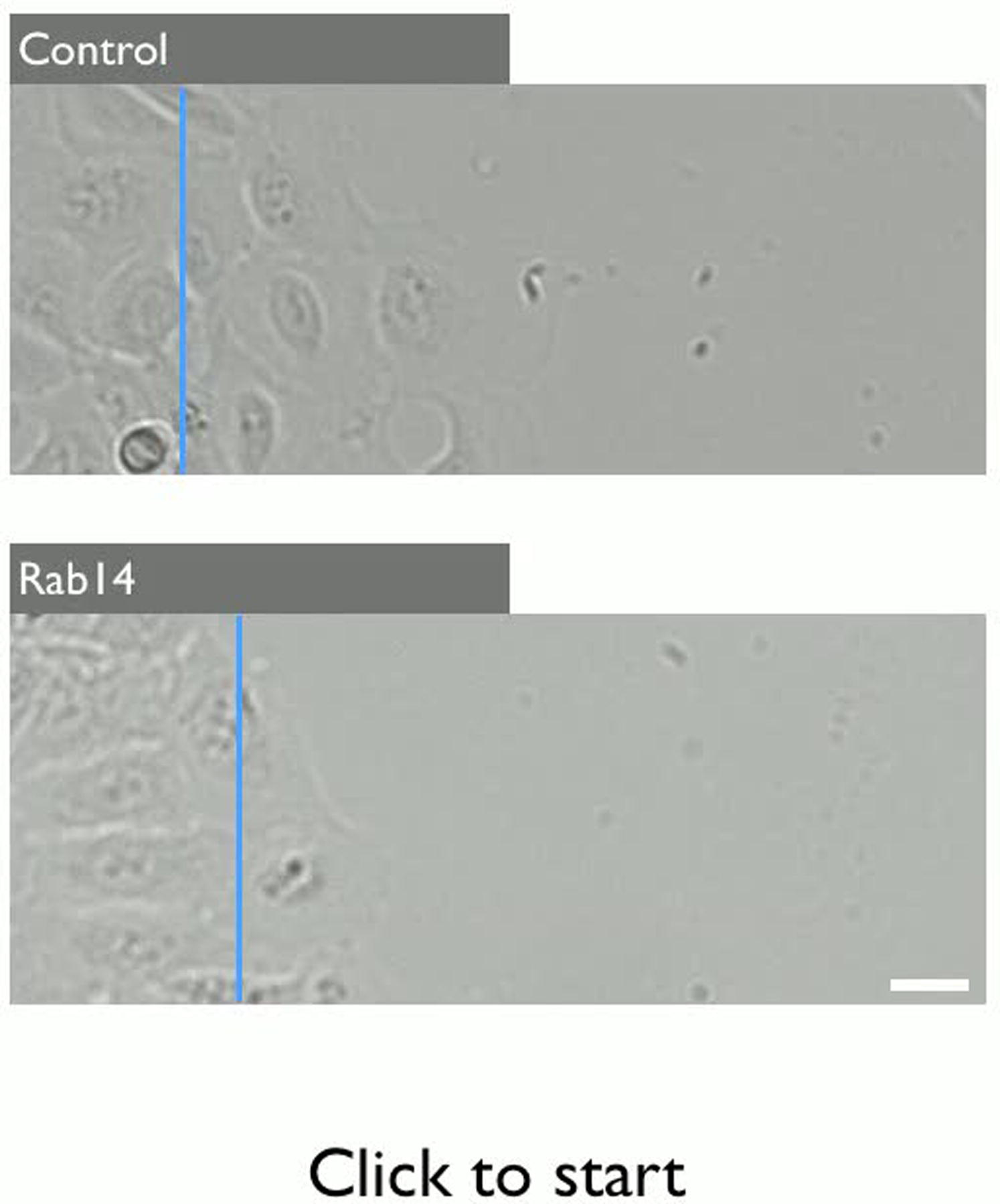

Supplement: Movie S2. Migration of Rab14-Depleted Cells — Enlarged movies showing the control and Rab14-depleted cells from the migration screen described in Figure 1. Images were taken every 5 min for 16 hr. The position of the wound edge at t0 is shown. [file mmc4.jpg]

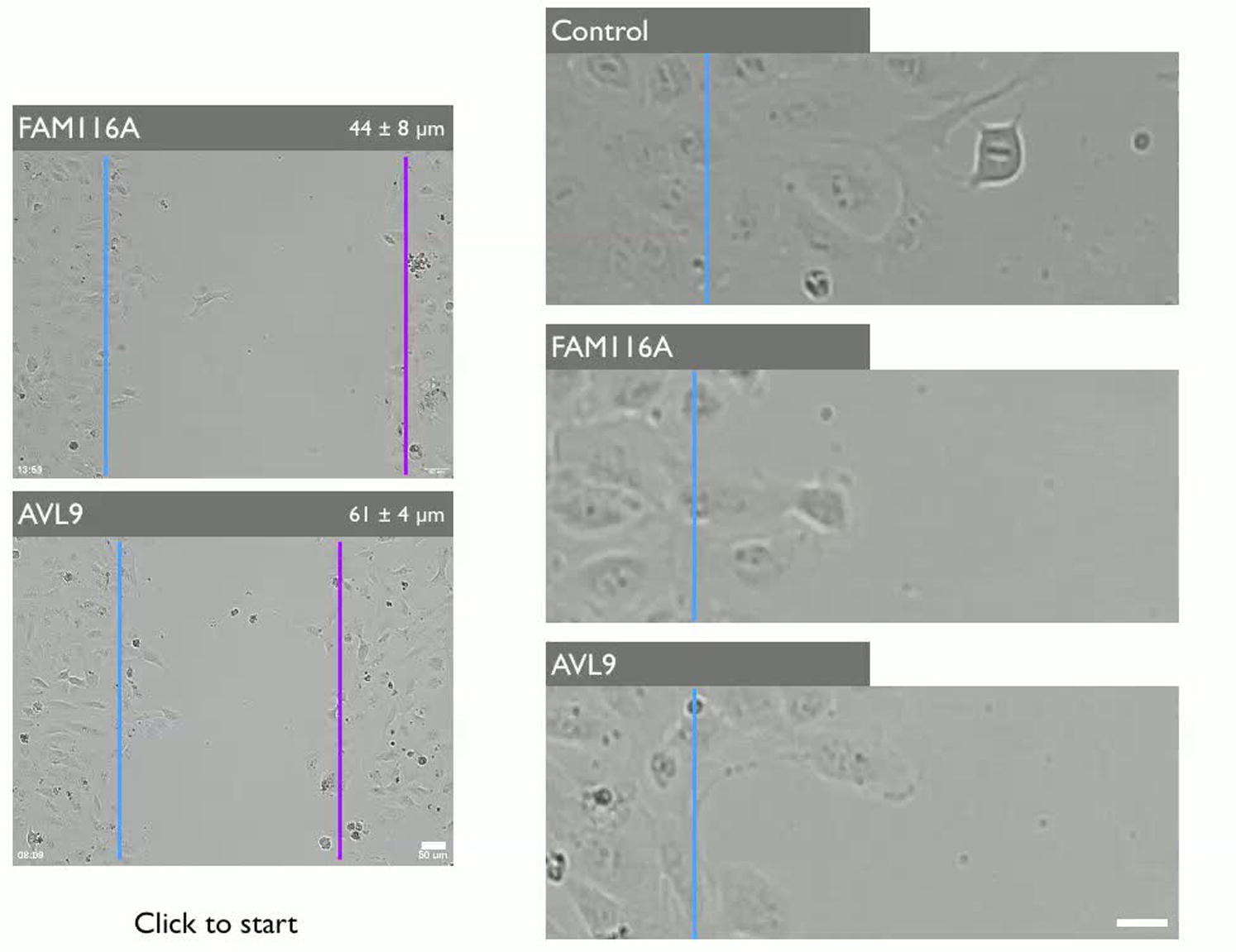

Supplement: Movie S3. Candidate Rab GEFs Required for Cell Migration — Movies showing the control, Avl9, and FAM116A-depleted cells from the migration screen described in Figure 2. Images were taken every 5 min for 16 hr. The position of the wound edges at t0 and 16 hr are shown. Migration was calculated from the difference in these values. The enlarged movies show more detail of the events at the wound edge. [file mmc5.jpg]

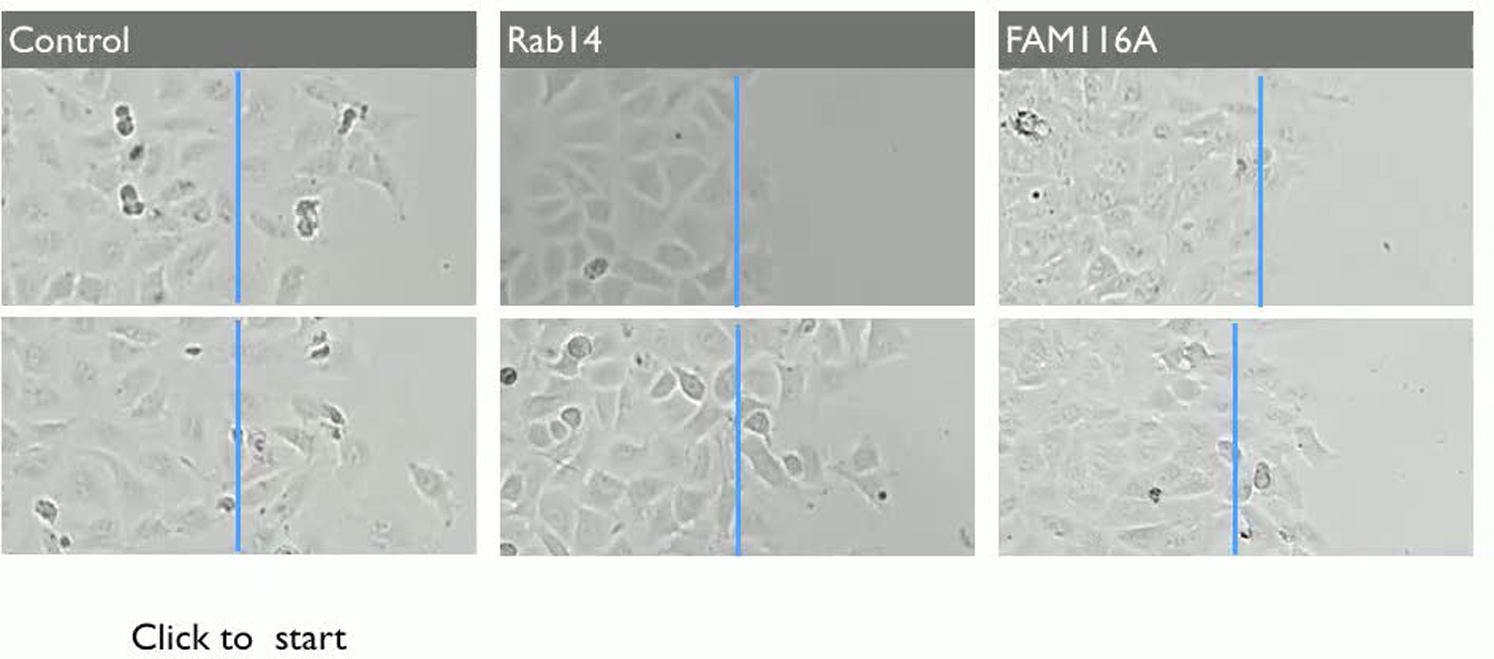

Supplement: Movie S6. Calcium Chelation Recovers the Migration Defect in Rab14-Depleted Cells — Enlarged movies showing the control and Rab14-depleted cells in the presence or absence of 1 mM EDTA treatment, as described in Figures S6 and S7. Images were taken every 5 min for 16 hr. [file mmc8.jpg]
